# Effects of biochar, dual inhibitor, and straw return on maize yield, soil physicochemical properties, and microbial system under fertilization conditions

**DOI:** 10.3389/fmicb.2025.1570237

**Published:** 2025-04-28

**Authors:** Zhanbo Wei, Xiaori Han, Yonghuan Wang, Lili Zhang, Ping Gong, Yuanliang Shi

**Affiliations:** ^1^College of Land and Environment, Shenyang Agricultural University, Shenyang, China; ^2^Institute of Applied Ecology, Chinese Academy of Sciences, Shenyang, China; ^3^Liaoning Agricultural Development Service Center, Shenyang, China

**Keywords:** fertilizers, inhibitors, biochar, straw returning to field, microorganisms

## Abstract

**Introduction:**

Although fertilizers play an important role in achieving high crop yields, improper nitrogen management and application measures have led to a series of ecological and environmental problems. Optimizing fertilization practices in agriculture is crucial for enhancing crop productivity while ensuring sustainable food production.

**Methods:**

This study aims to explore the effects of different fertilization regimes on crop yield, soil physicochemical properties, and microbial ecosystems. During the maize planting process, five fertilization treatments were applied: no fertilizer (CK); conventional fertilization (U), conventional fertilization with composite biochar (UB), conventional fertilization with urease/nitrification inhibitors (UI/NI), and conventional fertilization with straw return (UST).

**Results:**

The results indicate that maize yield under UI/NI treatment was higher than that under U treatment. The microbial community composition among the fertilization treatments had the same dominant species, but the relative abundance of species varied depending on the fertilization treatment; UB and UI/NI enhanced the role of dominant bacterial populations in the soil, while the UST treatment led to the formation of larger and more complex networks of soil bacterial communities.

**Discussion:**

This study provides scientific and theoretical support for the development and promotion of rational fertilizer application.

## 1 Introduction

Maize is grown worldwide and is a staple food for more than half of the global population (Yang et al., 2020). Farmers’ primary method for achieving higher yields is the excessive use of fertilizers, including nitrogen, phosphorus, and potassium (Kiran et al., 2022). However, in recent decades, the excessive use of pesticides and chemical fertilizers has severely degraded arable land, with soil organic matter content decreasing by 60% (Daniel and Scow, 2024). In particular, long-term application of inorganic fertilizers will also reduce microbial abundance and microbial catabolic activity (Su et al., 2025). Therefore, there is an urgent need to adopt environmentally friendly and ecofriendly strategies to reduce nutrient loss, improve efficiency, and sustainably enhance crop productivity. To solve these environmental problems, scientists have proposed a variety of environmental measures aimed at reducing nutrient loss and increasing crop yields Straw returning to the field, biochar application, and the use of stabilizers are currently the main technical methods for soil improvement.

The role of straw returning, a traditional method of agricultural recycling, is receiving increasing attention. Returning straw to the field can provide the soil with organic matter, increasing its fertility and biological activity (Wang et al., 2022). The decomposition of straw increases the number and diversity of microorganisms in the soil, improves the organic matter content of the soil, and enhances the soil structure and its ability to retain water and nutrients (Xu et al., 2018). Therefore, combining straw return with conventional fertilization helps to enhance soil sustainability and improve crop productivity.

In addition to straw returning to the field, another promising method for soil improvement is the application of biochar. Biochar is a carbon-rich material produced by pyrolyzing organic matter (such as straw or wood), characterized by high carbon content, a large specific surface area, low bulk density, high porosity, and strong adsorption capacity (Mohammadpour and Mir Mohamad Sadegh, 2020; Marcinczyk and Oleszczuk, 2022). Wen et al. (2022) found that the incorporation of biochar seems to have a positive effect on mycorrhizal fungi. Xie et al. (2022) found that the addition of biochar can significantly increase soil microbial biomass and crop yield relative to the application of nitrogen fertilizer alone and reduce the nitrogen application by 20% to achieve exact crop yield.

Nitrogen fertilizer stabilizers, including dicyandiamide nitrification inhibitors (NI) and urease inhibitors (UI), are considered key methods to address agricultural nitrogen pollution issues (Sha et al., 2020). Widely used commercial UI and NI, such as N-(n-butyl) thiophosphoryl triamide (NBPT), and 3,4-dimethylpyrazole phosphate (DMPP), play an important role in global agriculture (Tufail et al., 2022). The application of urease inhibitors in combination with urea can delay urea hydrolysis, providing plants with a greater opportunity to absorb nitrogen in this more energy-efficient form while reducing gaseous nitrogen losses (He et al., 2018). It can also affect soil microbial communities and biochemical processes, potentially influencing nitrogen dynamics in the soil (Zhang et al., 2022).

To assess the impact of fertilization strategies on agricultural production, five fertilization treatments were designed in this study: no fertilizer (CK), conventional fertilization (U), conventional fertilization with composite biochar (UB), conventional fertilization with composite (UI/NI), and conventional fertilization with composite straw return (UST). By comparing these fertilization models, the study aims to identify the most suitable fertilizer management methods, providing theoretical support for achieving sustainable agricultural development and ecological environmental protection.

## 2 Materials and methods

### 2.1 Experimental location and materials

Field experiments were carried out at Shenyang Farmland Ecosystem National Field Scientific Observation and Research Station (41°31′N, 123°24′E) in Shenyang, China. The experimental station is geographically located in the southern part of the Liaohe Plain, with an average elevation of 41 meters, an annual average temperature of 7°C–8°C, an annual active accumulated temperature above 10°C of 3,100°C–3,400°C, an annual total radiation of 120–135 kilocalories per square centimeter, a frost-free period of 147–164 days, and an annual precipitation of 650–700 millimeters. At the beginning of the test, the organic matter content in the soil was 17.2 g kg^−1^, the available nitrogen content was 134.94 mg kg^−1^, the available phosphorus was 38.84 mg kg^−1^, and the available potassium was 177.67 mg kg^−1^ with a pH of 6.85. Basic soil physical and chemical properties were determined before the experiment. The organic matter content in the soil was 17.2 g kg^−1^, a total N of 1.0 g kg^−1^, a total phosphate (P) of 0.4 g kg^−1^, a total potassium (K) of 22.8 g kg^−1^, the available N content was 134.94 mg kg^−1^, the available P was 38.84 mg kg^−1^, and the exchangeable K was 86.7 mg kg^−1^. The ammonium nitrogen (NH_4_^+^-N) was 4.85 mg kg^−1^, and the nitrate nitrogen (NO_3_^−^-N) was 6.49 mg kg^−1^. Soil bulk density (0 to 20 cm) was 1.51 g cm^−3^, and the pH was 6.85. The agricultural system grows only one season of corn a year.

A completely randomized design was used with 5 treatments and 3 replicates. The area of each plot was 9 m^2^ (3 m × 3 m). The treatments were (1) no fertilizer (CK); (2) conventional fertilization (U) (150 kg N/ha, 52 kg P/ha, and 105 kg K/ha); (3) conventional fertilization and biochar (UB) (4,111 kg biochar ha^−1^) (Ajayi and Horn, 2016); (4) conventional fertilization and inhibitor (UI/NI) (The amount of NBPT (N- (n-butyl) thiophosphoric acid triamide) added was 1% of the fertilizer weight and 2% DMPP (3,4-dimethylpyrazole phosphate) added was 2% of the fertilizer weight) (Lv et al., 2024); (5) urea and straw (US) (full return of straw to the field). N was provided by urea, P by heavy superphosphate, and K by potassium sulfate. When the land was prepared into ridges, the whole layer fertilization method was adopted, with a single application of fertilizer. Weeds, pests, and diseases were controlled in accordance with farming practices.

Maize 12-leaf stage (V12 stage) marked the transition from vegetative growth to reproductive growth, when crops entered the rapid growth period and nutrient absorption rate reached the peak. During this period, the peak release of fertilization (such as urea, phosphate, potassium) has passed, and soil residual nutrients and crop absorption have reached a dynamic balance. Therefore, soil samples were collected using special soil drilling tools from the 12-leaf collar (50 days after planting) of maize, and each soil sample was split into three subsamples. One subsample was air-dried, sieved with a 2 mm sieve and analyzed for soil pH, ammonium (NH_4_^+^), nitrate (NO_3_^−^), and organic matter. The second subsample was kept moist to measure soil enzyme activity. The remaining sample was delivered to the laboratory and stored at −80°C refrigeration for detection of microbial communities. Soil pH was measured in a 1:2.5 soil/water suspension using a combination electrode. Soil inorganic nitrogen (NH_4_^+^-N and NO_3_^−^-N) was extracted with 2 mol L^−1^ KCl and determined on a continuous flow analyzer (AA III, Norderstedt, Germany). Soil organic matter content was measured using a standard solution titration of ferrous sulfate (Bao, 2000). Soluble humus was extracted from a 0.1 mol L^−1^ mixed solution of sodium pyrophosphate and sodium hydroxide, and the total amount of humic acid (HA) and fulvic acid (FA) was determined. PQ = HA/ (HA + FA), which indicates the soil degree of humification. In accordance with Dux’s law, the suspensions were graded to determine the aggregates based on the settling time of microaggregates with different diameters. Urease activity was determined according to the method described by Tabatabai (1994). A specified amount of urea solution was added to the soil sample and incubated at 37°C for 5 h. The residual Urea-N was measured, and the amount of hydrolyzed urea per unit time was calculated to evaluate soil urease activity, expressed as mg kg^−1^ h^−1^. The activity of the soil enzymes was measured using an ELISA kit provided by the Shanghai Hengyuan Biological Technology Co. Ltd. (Shanghai, China).

Soil aggregates were divided into large macroaggregates (>2 mm), small macroaggregates (0.25–2 mm), and micro-aggregates (<0.25 mm) using the wet sieve method (Wright, 2009). The relevant soil aggregate indexes [particle sizes larger than 0.25 mm (R_0.25_), the mean weight diameter (MWD) and the geometric mean diameter (GMD)] are as follows (Hu et al., 2021):


(1) $$R_{0.25}=100\frac{\sum_{i=3}^{n}wi}{w}$$

(2) $$MWD=\frac{\sum_{i=3}^{n}d_{i}wi}{W}$$

(3) $$GMD=\exp\left(\frac{\ln d_{i}\sum_{i=1}^{n}\frac{w_{i}}{w}}{w}\right)$$

In Equations 1–3, R_0.25_ is the proportion of the dry weight of soil aggregates larger than 0.25 mm; d_i_ is the average diameter of the *i*th size fraction of the aggregate; W_i_ is the mass fraction of aggregates remaining on *i*th sieve; n is the number of the total series of the sieve.

### 2.2 Statistical analyses

Various software and indicators were used to visually analyze the results on soil physicochemical properties, urease activity, and the structure of the soil urease microbial community. Statistical analysis of the basic physicochemical properties, urease activity, and phylum-level abundance among different treatments was conducted using SPSS 20 (SPSS Inc., IL, United States). Mean differences among treatments were analyzed using the least significant difference (LSD) test at a 5% probability level. Origin software (Version 2021b, OriginLab Corporation, MA, United States) was used for graphical presentation of the data. Community composition abundance was visualized using the “ggplot2” package in R. Shannon and Chao1 index were calculated by “vegan” package in R and plotted with “ggplot2.” PCoA is a method used to analyze differences between communities, similar to the multidimensional scaling typically used to represent *β*-diversity. Principal coordinate analysis (PCoA) and classical correspondence analysis (CCA), based on the Bray–Curtis distance, were used to evaluate the soil microbial functional guild structure under different treatments and their correlations with environmental factors. Network analysis is often used to correlate species abundance information between samples to obtain species coexistence, constructing a symbiotic network based on Spearman correlation using “WGCNA” package. The network parameters were calculated with “igraph” package in R, and the network graphs were visualized with Gephi (ver. 0.10.1). The Mantel test correlation plot was generated using ChiPlot^1^.

## 3 Results

### 3.1 Changes in soil properties

**Figure 1:**
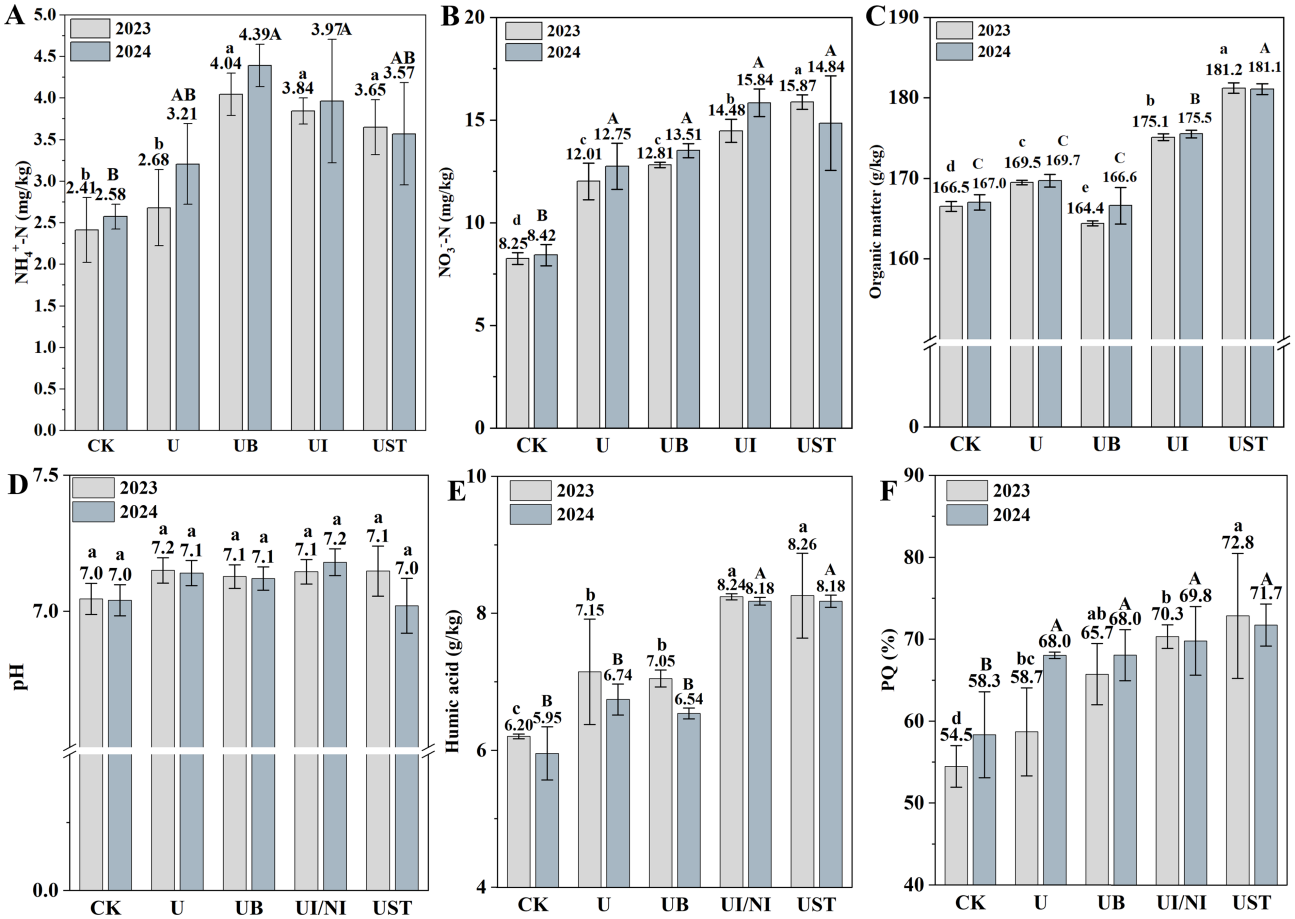
NH_4_^+^-N **(A)**, NO_3_^−^-N **(B)**, organic matter **(C)**, pH value **(D)**, Humic acid content **(E)**, PQ (%) values **(F)** in soil. The values are given as averages, and the error line represents the standard deviation. The significant differences in fertilization treatment in 2023 and 2024 are represented by lowercase and uppercase letters, respectively (*p* < 0.05).

Changes in fertilizer treatment altered the soil nutrient variables (Figure 1). In 2023, the NH_4_^+^-N content of UB, UI/NI and UST was higher than U (Figure 1A). The NO_3_^−^-N content of UI/NI and UST was higher than U (Figure 1B). After treatment with UB, UI/NI, and US, there was no significant difference in soil pH, but it remained within the normal range (7.0–8.0) (Figure 1D). UI/NI and UST significantly higher than soil organic matter and HA content compared to U (Figures 1C,E). The PQ value indicates soil humification. In general, the humification process was significantly promoted by straw returning to the field (Figure 1F).

Fertilization treatment affects soil aggregate composition. Large aggregates (>2 mm) were the most prominent in the soil, followed by small aggregates (0.25–2 mm) and microaggregates (<0.25 mm) (Supplementary Table S1). The content of small aggregates in UB and UST did not show significant changes compared to U. Fertilization treatment significantly affected soil enzyme activity, but the degree of influence on enzyme activity was different (Table 1). The activities of cellulase, hemicellulase and *β*-glucosidase in fertilization treatment were higher than those in CK treatment. The nitrogen cycling enzyme (urease and nitric reductase) activities in soil treated by UB and UST were higher than treated by U.

**Table 1:** Enzyme activity in soil under different treatments.

Treatment	Cellulase (mg/d/g)		Hemicellulase (mg/d/g)		β-glucosidase (μmol/d/g)		N-acetyl-β-glucosaminidase (μmol/d/g)		Urease (μg/d/g)		Nitrate reductase (nmol/d/g)	
	2023	2024	2023	2024	2023	2024	2023	2024	2023	2024	2023	2024
CK	0.34 c	0.33 c	854.44 c	904.85 c	10.54 b	10.56 c	0.11 b	0.11 a	64.94 d	71.40 c	37.46 d	40.36 d
U	0.38 b	0.37 b	968.58 b	985.30 b	13.10 a	12.30 b	0.21 a	0.23 a	88.12 c	86.16 b	87.54 c	86.78 c
UB	0.39 ab	0.38 ab	1034.69 ab	999.00 ab	14.50 a	16.18 a	0.25 a	0.24 a	97.49 ab	88.60 b	88.36 c	111.13 b
UI/NI	0.41 a	0.38 ab	1986.22 a	990.18 b	13.98 a	17.27 a	0.23 a	0.31 a	91.80 bc	89.17 b	114.74 b	131.34 ab
UST	0.39 ab	0.40 a	1035.71 ab	1010.89a	15.50 a	16.43 a	0.27 a	0.29 a	101.63 a	102.29 a	138.80 a	154.25 a

One-way ANOVA based on Duncan’s multi-range test (*p* < 0.05) showed no significant difference in the mean values after the same letter within each column.

### 3.2 Impact of fertilization treatment on maize yield

The yield of U treatment was higher than that of CK treatment (Table 2). In the fourth year, the corn yield of U treatment increased the most than CK, reaching 38.01%. In the fertilization treatment, UI/NI had the highest yield increase, which increased the yield by 17.71, 11.86, 23.80 and 13.02% compared with U in 2021–2024, respectively (*p* < 0.05). Similarly, the maize yield of UB was 0.85 to 13.42% higher than that of U. The fertilization treatment with straw return did not show significant differences from the U, but in the third year, it exhibited the highest yield increase, with an increase of 14.22% (*p* < 0.05).

**Table 2:** Maize production in 2021–2024.

Treatment	2021	2022	2023	2024
CK	864.06 ± 27.59 c	1057.78 ± 33.90 c	1120.00 ± 62.09 d	1044.01 ± 79.43 c
U	1049.07 ± 23.76 b	1223.70 ± 42.53 b	1298.37 ± 80.38 c	1440.90 ± 185.08 b
UB	1058.61 ± 26.50 b	1234.07 ± 63.93 b	1472.59 ± 29.33 b	1456.00 ± 37.76 b
UI/NI	1234.90 ± 36.30 a	1368.89 ± 25.40 a	1607.41 ± 24.77 a	1641.51 ± 81.17 a
UST	1100.92 ± 3.85 b	1327.41 ± 52.88 ab	1482.96 ± 14.67 b	1449.97 ± 63.57 b

One-way ANOVA based on Duncan’s multi-range test (*p* < 0.05) showed no significant difference in the mean values after the same letter within each column.

### 3.3 Composition, diversity, and structure of microbial communities under different fertilization treatments

**Figure 2:**
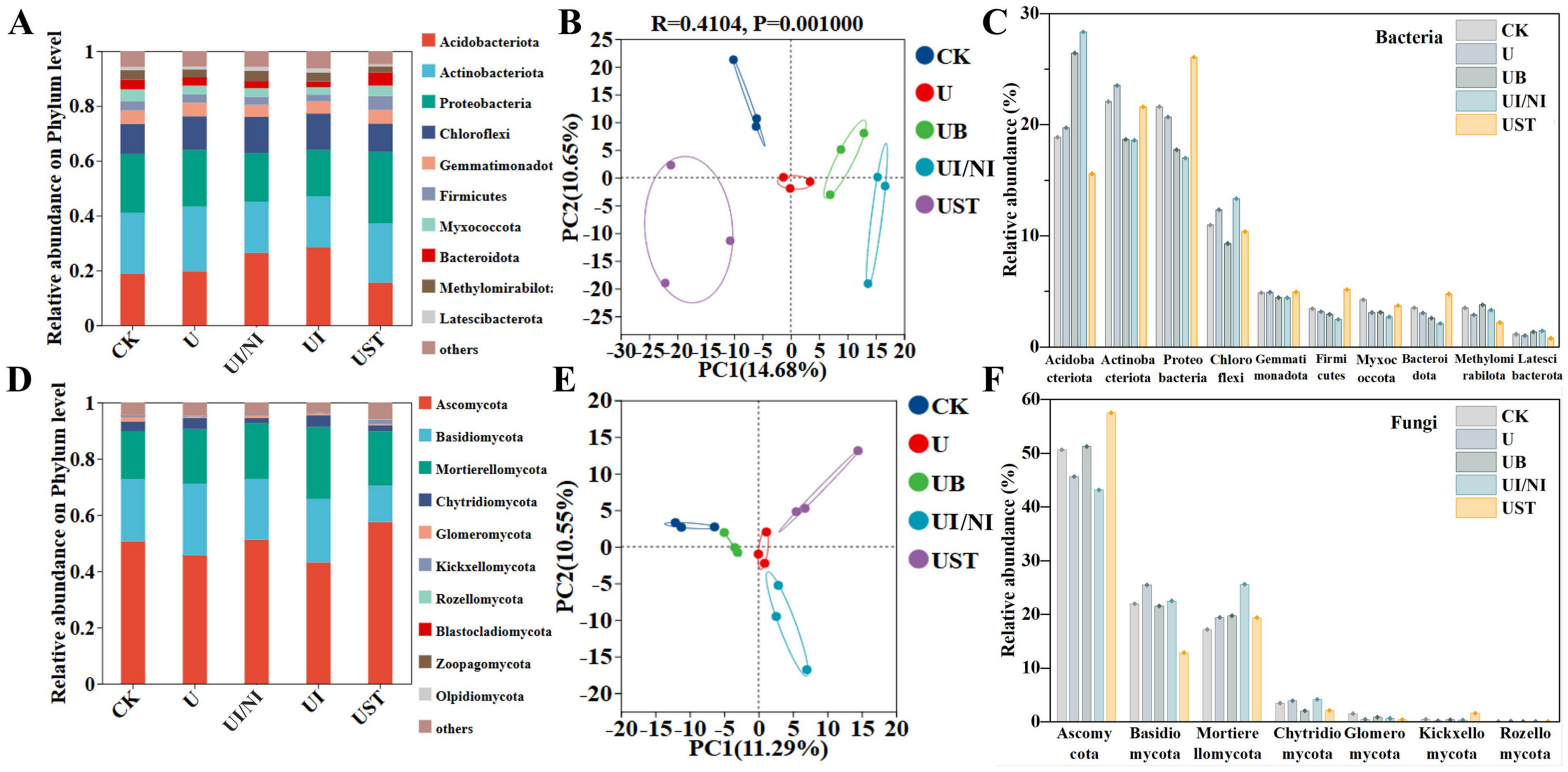
Functional composition of bacteria **(A)** and fungi **(D)** in the farmland ecosystem under different fertilization treatments; genus-level principal coordinate analysis of soil bacteria **(B)** and fungi **(C)** communities based on Bray-Curtis distance; hierarchical clustering analysis of bacteria **(E)** and fungi **(F)** communities based on operational taxonomic units (OTUs).

**Figure 3:**
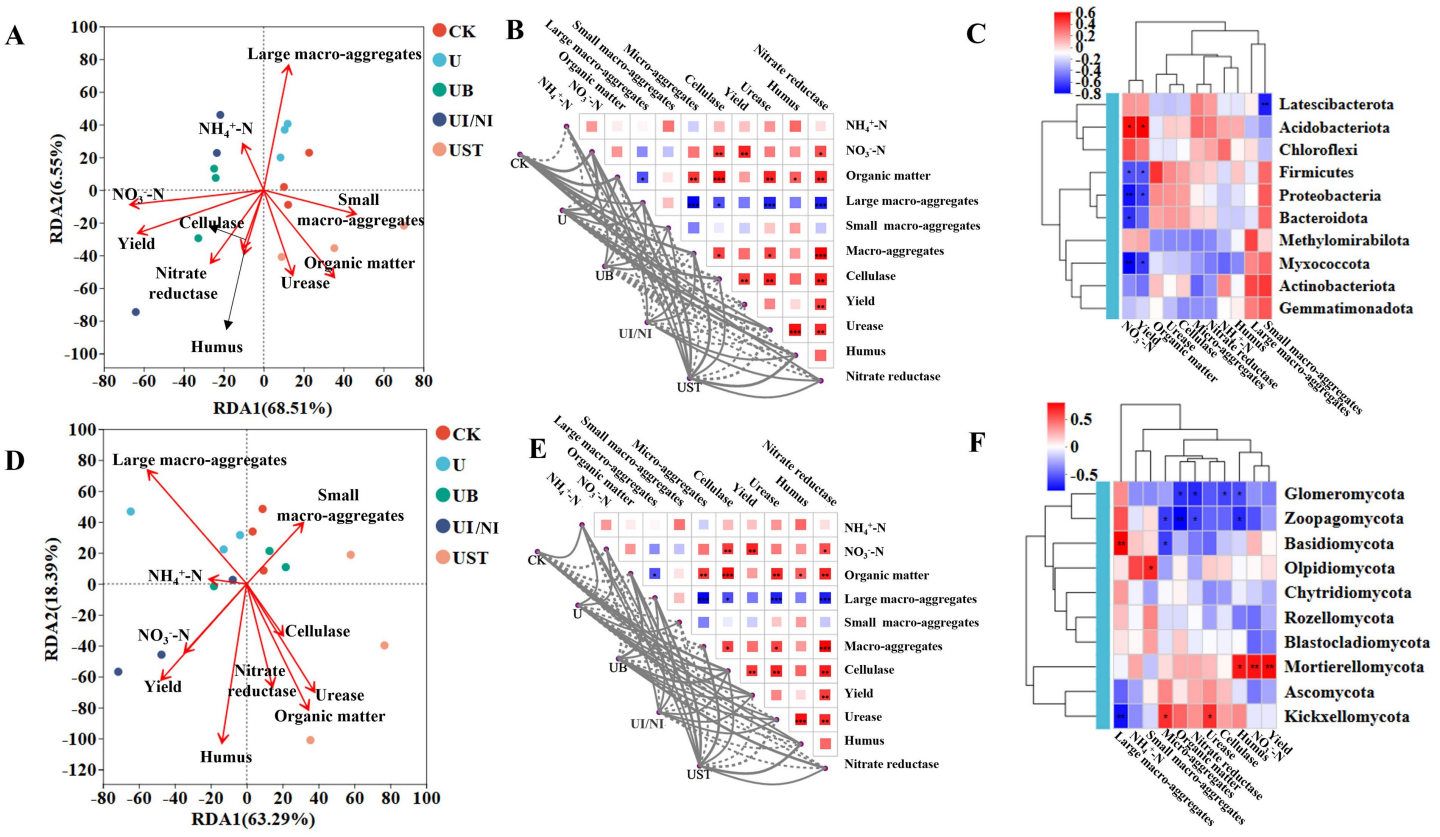
Correlation analysis of bacterial functions related to soil characteristics **(A)** Mantel test results **(B)** and correlation Heatmap **(C)**; Correlation analysis of fungal functions related to soil characteristics **(D)** Mantel test results **(E)** and correlation Heatmap **(F)**. Line thickness: the correlation between community and environmental factors was drawn with Mantel “*r*” (absolute value of *r*); the asterisks in the color block represent significance, *0.01 < *p* ≤ 0.05, **0.001 < *p* ≤ 0.01, ***p ≤ 0.001.

Fertilization treatment affected the relative abundance of bacteria and fungi. Acidobacteriota, acmobaeteriota, proteobacteria, and chloroflexi dominated the bacterial community (Figure 2A). Compared with U, the relative abundance of acidophilus higher than UB and UI/NI (Figure 2C). UST had the most obvious effect on the relative abundance of proteobacteria (Figure 2F). The fungal communities consisting of ascomycota, basidiomycota, mortierellomycota, chytridiomycota, glomeromycota were dominant (Figure 3D). The relative abundance of ascomycetes in UB was higher than that in U. Bacteria (25.33%) (Figure 2B) and fungi (21.84%) (Figure 2E) showed different changes in community structure. There was no significant difference in Chao index and fungal *α* diversity among bacteria under different fertilization treatments (Supplementary Figure S1).

### 3.4 Relationship between microbial function and soil characteristics under different fertilization treatments

Mantel analysis was used to evaluate the relationship between soil physical and chemical properties, yield, urease activity, and functional combinations of bacteria and fungi (Supplementary Table S2). The correlation between soil physicochemical factors and microbial functional groups varied (Figures 3A,B). The content of ammonium nitrogen and nitrate nitrogen in the soil was positively correlated with the function of actinobacteriota (Figure 3C). There is a strong correlation between urease, nitrate reductase and organic matter (Figure 3C). The contents of organic matter and humus in the soil were significantly correlated with fungi (Figures 3E,F).

**Figure 4:**
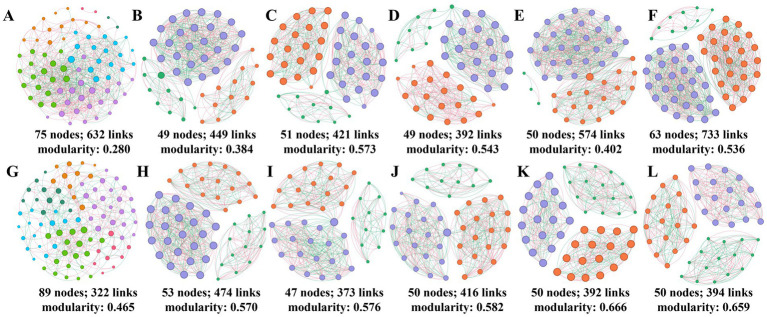
Symbiotic network of soil bacteria under different treatments **(A)**, CK **(B)**, U **(C)**, UB **(D)**, UI/NI **(E)**, and UST **(F)**. The symbiotic network of soil fungi under different treatments **(G)**, CK **(H)**, U **(I)**, UB **(J)**, UI/NI **(K)**, and UST **(L)** was comprehensively analyzed.

Because microbial groups tend to occur together rather than separately, we assessed the effects of different fertilization treatments on soil microbial co-occurrence networks (Figure 4). Within the overall module, the soil bacterial community had more nodes and the fungal community had more edges. Compared with U-treated networks, UST soil bacterial populations produced larger and more complex networks with the most network nodes and edges, while there was no significant difference between U, UB, and UI/NI treatments (Supplementary Figure S2).

## 4 Discussion

### 4.1 Fertilizer management practices improve soil nutrient and enzyme activity, leading to increased crop yield

Conventional fertilization combined with biochar, inhibitors, or straw returning can significantly change soil nitrogen content. The contents of NH_4_^+^-N and NO_3_^−^-N in soil under the treatment of biochar and straw were higher than that of U, which may be related to the promotion of soil organic matter and microbial activity. Biochar has a significantly higher void volume, surface area, and surface negative charge. At the same time, it also has a very large cation exchange capacity (Cooper et al., 2020). These properties help to extend the retention time of cations and promote the slow release of nutrients from biochar, thus contributing to improved land quality. Biochar can retain NH_4_^+^-N in the soil by cation exchange through acid functional groups on its surface, such as carboxyl and hydroxyl groups. However, straw return to the field provides more nitrogen sources by increasing organic matter, which directly promotes the generation of HA and thus promotes the mineralization of nitrogen (Xu et al., 2018). Keeping soil pH stable is essential for nutrient uptake by plants and growth of soil microorganisms (Zhang et al., 2020). Biochar, inhibitors, and straw returning to the field have less impact on soil pH, which allows these treatments to avoid excessive acidification or alkalization while maintaining soil nutrition and microbial activity.

Fertilization promotes the growth of crops, and the crop residues and root secretions produced during plant growth are decomposed into usable small molecules of glucose under the action of cellulase and hemicellulose. *β*-glucosidase releases glucose or glucose derivatives by hydrolyzing β-glucoside bonds. These all provide an energy source for microbial activity (Greenfield et al., 2020). Meanwhile, it plays a crucial role in the decomposition of litter and its conversion to soil organic matter. Urease converts soil organic nitrogen into inorganic nitrogen that can be used by plants and microorganisms (Fisher et al., 2017; Jiang et al., 2023). The results showed that UST treatment had higher soil urease activity, which may be due to the increase of soil microbial metabolism and stimulation of soil microbial activity, as well as the change in microbial community composition after straw returning to the field. Contrary to expectations, the addition of the inhibitor did not reduce soil urease activity, possibly because sampling occurred after the half-life of the inhibitor had passed, indicating that the inhibitor was not present in the soil at this time. UB, UI/NI, and UST had higher maize yields by providing adequate nutrients, inhibiting nitrogen loss, and increasing fertilizer use efficiency.

### 4.2 Differing composition, diversity, and community structure of soil microbial functions under different fertilization treatments

Biochar acts as a soil amendment; it can improve soil organic matter content and water retention ability (Jin et al., 2024). With the addition of biochar, the growth of Ascomycota can promote the decomposition of plant residues, lignin, cellulose, and other organic matter in the soil, releasing usable carbon sources. Biochar can also increase the activity of denitrifying agents by promoting the growth of filamentous bacteria (Krause et al., 2018). Unlike the short-term effects of this study, in a 6-year experiment, it was found that the chao1and Simpson indices of soil bacterial communities decreased significantly under long-term continuous application of biochar (Peake et al., 2014). This was because under the action of biochar for a long time, the structure of soil bacteria develops in a specific direction, which eventually leads to a decrease in diversity and abundance (Xu et al., 2022). Therefore, the long-term dynamic observation of soil microbial communities in soil ecosystems should be strengthened.

The relative content of acidophilus in soil under UI/NI treatment is higher than that of UB and UST. On the one hand, it may be because inhibitors (such as UI) may promote the growth and reproduction of acidophilus by changing the conversion mode of nitrogen (Wu et al., 2023). On the other hand, it may be because the biochar and straw added to the soil increased the organic carbon content of the soil. Acidobacteria are oligotrophic bacteria and prefer the growth of soil with low organic carbon content (Wang et al., 2022). The inhibitory effect of UI/NI treatment on actinomycetes and chytridomycetes may be related to changes in nitrogen and carbon sources in the soil and the effects of microbial competition (Guan et al., 2023). Ascomycetes often feed on decomposing plant residues, and the addition of straw provides more organic matter. This provides them with a rich source of nutrients (Maarastawi et al., 2019).

### 4.3 Effects of taxon–taxon network connections of soil bacteria and fungi on the growth environment of maize

In general, a more complex microbial network is strongly positively associated with versatility and represents stronger cooperative behavior between microorganisms (Chen et al., 2023). Straw returning to the field adds a large amount of organic matter (such as cellulose, lignin, etc.) to the soil and provides a rich source of nutrients for microorganisms. Different metabolites such as organic acids produced by microbial decomposition of straw can promote the growth of specific microbial communities, thus promoting the diversity and complexity of soil microbial communities. A more complex network structure is formed (Zhao et al., 2024). These complex microbial networks can enhance the health of ecosystems and reduce susceptibility to disease. Especially in agricultural systems, through complex networks, microbial communities can engage in synergistic interactions that defeat pathogens, enhance plant immune responses, and help maintain soil health (Wang et al., 2024). For example, certain beneficial microorganisms in the rhizosphere (such as mycorrhizal fungi) form a reciprocal relationship with the plant, which not only improves nutrient uptake, but also provides resistance to root pathogens (Qiao et al., 2024). These findings suggest that changes in microbial interaction networks, particularly the hubs and connectors of some key modules, may be one of the key factors in significantly promoting straw decomposition and nitrogen release. What’s more, the results showed that UST treatment alters the soil microbial community, promoting the proliferation of actinomycetes, proteobacteria and ascomycetes, which also play an important role in breaking down organic matter such as plant residues, lignin and cellulose, converting organic nitrogen into inorganic nitrogen forms, and promoting the growth of the soil (Zhang et al., 2017). In this way, it participates in the carbon assimilation and nitrogen cycle in the soil, promoting the recycling of nutrients and helping to maintain the stability of the ecosystem (Lu et al., 2023).

## 5 Conclusion

This study investigated the effects of straw returning, biochar, and inhibitor fertilization on soil physicochemical properties in agroecosystems, and revealed that soil bacteria and fungi communities were affected by various fertilization treatments. U, UB, UI/NI, and UST improved the physical and chemical properties of the soil: UI/NI has a higher yield and the contents of NH_4_^+^-N, NO_3_^−^-N, and HA in the soil increased the most significantly; UST was beneficial to the increase in soil organic matter content. Soil pH under several treatments remained in the stable range. However, although fertilization did not change the diversity of bacterial and fungal communities, there were different effects on the relative abundance of soil microbial communities. UB and UI/NI had higher relative abundance of acidomycetes, and UST increased the relative abundance of microorganisms with cellulose and lignin decomposition functions. Specifically, the study revealed that rational fertilization plays an important role in maize yield, soil microbial networks, carbon/nitrogen cycling enzyme levels, and soil nutrient binding. The initial physical and chemical properties of soil and the mechanism of long-term application of additives to promote crop growth and improve soil properties still need to be further explored. In addition, fertilization measures under various agro-climatic conditions, different crops and soil types should be studied in the future to develop effective fertilization strategies.

## Data Availability

The original contributions presented in the study are included in the article/Supplementary material, further inquiries can be directed to the corresponding authors.

## Footnotes

^1^ https://www.chiplot.online/
