# Supplementary material for: Effects of biochar, dual inhibitor, and straw return on maize yield, soil physicochemical properties, and microbial system under fertilization conditions
Source: Front Microbiol. 2025 Apr 28;16:1570237. doi: 10.3389/fmicb.2025.1570237 (PMC12066768; doi:10.3389/fmicb.2025.1570237)
Supplement: Supplementary file 1 [file Table_1.docx]

**Figure S1 Comparison of α diversity in functional communities of bacteria (A: Chao1, B: Simpson) and fungi (C: Chao1, D: Simpson)**


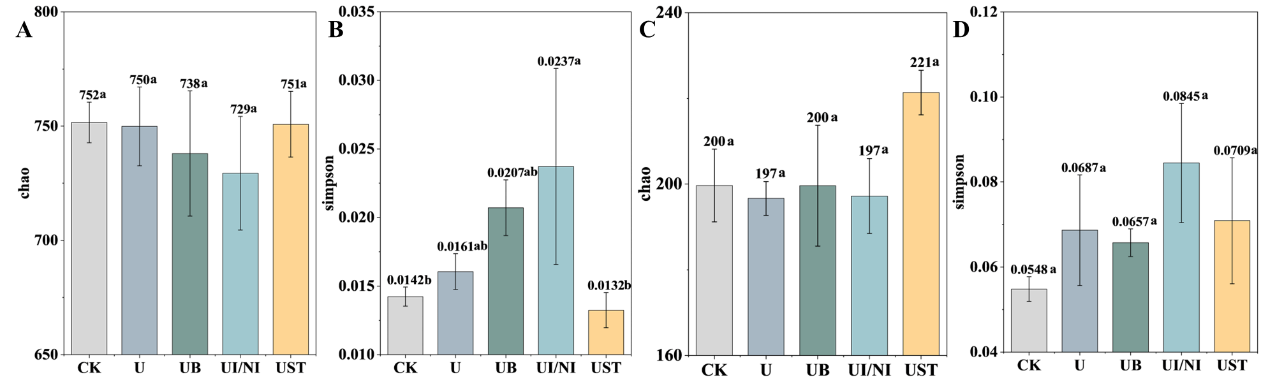


**Figure S2 Degree and intermediate centrality of nodes in soil bacterial (A) and fungal (B) networks under different fertilization treatments (log10 scale). Nonparametric tests were performed to assess the difference between the two topological indices in the four soil networks.**


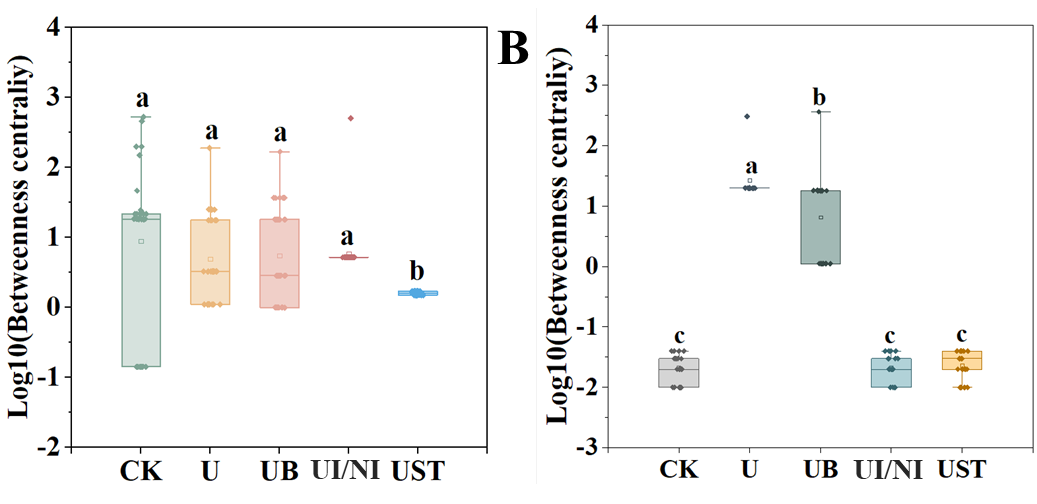


**Figure S3 Changes in the abundance of bacteria (A) and fungi (B) at the phylum level.**


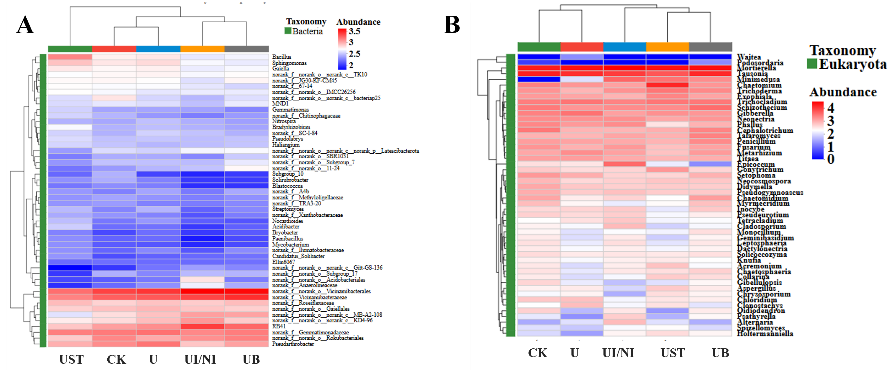


**Table S1. Soil aggregate size distribution and stability under different fertilization treatments**

| Treat-ment | **Aggregate proportion** | | | | | | | | | | | |
| --- | --- | --- | --- | --- | --- | --- | --- | --- | --- | --- | --- | --- |
|  | 2023 | | | Stability index | | | 2024 | | | Stability index | | |
|  | >2 mm | 0.25–2 mm | ＜0.25 mm | *R_0.25_* | *MWD* | *GMD* | >2 mm | 0.25–2 mm | ＜0.25 mm | *R_0.25_* | *MWD* | *GMD* |
| CK | 56.3 a | 20.7 ab | 23.1 b | 76.94 a | 3.08 a | 1.57 a | 56.1 a | 21.8 a | 22.1 b | 77.88 a | 3.08 a | 1.60 a |
| U | 55.9 a | 23.5 a | 20.6 b | 79.39 a | 3.08 a | 1.65 a | 54.8 ab | 22.8 a | 22.5 b | 77.51 a | 3.02 a | 1.55 a |
| UB | 52.0 b | 21.2 ab | 26.8 a | 73.18 b | 2.87 b | 1.36 b | 52.4 ab | 19.9 a | 27.7 a | 72.27 ab | 2.88 ab | 1.34 b |
| UI/NI | 50.7 b | 20.4 b | 28.9 a | 71.07 b | 2.80 bc | 1.27 b | 51.0 ab | 21.1 a | 27.9 a | 72.10 b | 2.82 b | 1.30 b |
| UST | 48.7 c | 21.9 ab | 29.4 a | 70.62 b | 2.72 c | 1.22 b | 50.0 b | 21.5 a | 28.5 a | 71.51 b | 2.78 a | 1.27 b |

Note: *R_0.25_* is the aggregate index for water-stable aggregate >0.25 mm; *MWD* is the mean weight diameter; *GMD* indicates the geometric mean diameter. One-way ANOVA based on Duncan’s multi-range test (*P* < 0.05) showed no significant difference in the mean values after the same letter within each column.

**Table S2 Correlation coefficient between soil properties and functional guild structure of bacteria and fungi in different agroecosystems based on Mantel test (r)**

|  | bcteria | | fungi | |
| --- | --- | --- | --- | --- |
|  | r | P | r | P |
| NH_4_^+^-N | 0.215 | 0.060 | 0.169 | 0.079 |
| NO_3_^-^-N | 0.102 | 0.201 | 0.247 | 0.027 |
| organic matter | 0.101 | 0.247 | 0.107 | 0.197 |
| large macro-aggregates | 0.120 | 0.224 | -0.009 | 0.466 |
| small macro-aggregates | 0.128 | 0.195 | 0.149 | 0.117 |
| micro-aggregates | 0.169 | 0.171 | 0.095 | 0.209 |
| humus | 0.101 | 0.253 | 0.122 | 0.161 |
| yield | -0.163 | 0.870 | 0.149 | 0.164 |
| urease | 0.113 | 0.213 | 0.097 | 0.209 |
| cellulase | 0.226 | 0.067 | 0.176 | 0.076 |
| nitrate reductase | 0.326 | 0.006 | 0.173 | 0.086 |
